# Diagnostic Accuracy of Endometrial Sampling Methods for Determining Histologic Type and Grade in Endometrial Cancer: A Retrospective Cohort Study

**DOI:** 10.7759/cureus.95428

**Published:** 2025-10-26

**Authors:** Dina Gumin, Avishalom Sharon, Susana Mustafa Mikhail, Inshirah Sgayer, Raneen Abushqara, Lior Lowenstein, Ala Aiob

**Affiliations:** 1 Obstetrics and Gynecology, Galilee Medical Center, Nahariya, ISR; 2 Obstetrics and Gynecology, Bar Ilan University Faculty of Medicine, Safed, ISR

**Keywords:** d&c, histologic concordance, pipelle biopsy, preoperative diagnosis, sampling accuracy

## Abstract

Introduction: Accurate preoperative diagnosis of histologic subtype and tumor grade is essential for optimal treatment planning. This study evaluated the diagnostic accuracy of endometrial sampling and compared it with hysteroscopy, dilation and curettage (D&C), and Pipelle biopsy in identifying the histologic subtype and tumor grade of endometrial carcinoma.

Materials and methods: This retrospective, single-center study analyzed data from 188 women with endometrial carcinoma who underwent primary hysterectomy following initial endometrial sampling at Galilee Medical Center (2010-2024). Patients were categorized by sampling method, and histologic concordance with final hysterectomy specimens was evaluated using Kappa statistics.

Results: Overall, histologic concordance for endometrial sampling for determining histologic type and grade in endometrial cancer was 83%. Hysteroscopy showed the highest accuracy for histologic subtype (91%) and tumor grading (76.9%), followed by D&C and Pipelle biopsy. Kappa analysis revealed moderate concordance for hysteroscopy (κ = 0.729) and D&C (κ = 0.731), with Pipelle biopsy showing weaker concordance (κ = 0.441). Hysteroscopy was significantly more accurate than Pipelle in detecting tumor grade (p = 0.017).

Conclusion: Endometrial sampling is moderately accurate for determining histologic type and grade in endometrial cancer. Hysteroscopy offers the highest precision for preoperative diagnosis, particularly in assessing histologic type and grade, supporting its role as the preferred diagnostic method. Future studies integrating molecular markers with histologic evaluation hold promise for improving diagnostic accuracy and optimizing treatment outcomes.

## Introduction

Endometrial cancer ranks as the sixth most common cancer among women globally and is the most frequently diagnosed gynecologic cancer in developed countries [1,2]. Over recent decades, endometrial cancer rates have markedly increased, likely driven by the rising prevalence of obesity and metabolic syndrome in these regions [1-4]. Forecasts suggest that endometrial cancer incidence will continue to rise in the coming years [5]. Given these trends, early diagnosis and tailored treatment approaches are essential. Currently, initial assessments for suspected endometrial cancer generally involve endometrial sampling techniques, such as curettage, hysteroscopic-guided curettage, or biopsy [6]. These methods detect invasive diseases and yield critical insights into molecular abnormalities, histological subtypes, and tumor grade, aiding in risk stratification [7-9]. Traditionally, Bokhman's histopathological classification system divides endometrial cancer into estrogen-sensitive, low-grade type I, and estrogen-independent, high-grade type II cancers [10]. However, this classification has been increasingly questioned with recent discoveries of molecular markers, including p53 mutations, mismatch repair deficiencies, and POLE mutations [7,11,12]. Despite these advancements, histological subtyping and grading remain integral to clinical practice for risk assessment before surgery [6,11,13]. Numerous studies have noted discrepancies between preoperative and postoperative tumor histology [14], which may lead to suboptimal treatment, as missing high-risk cancers have been linked to poorer outcomes in retrospective cohort studies [15]. There is a lack of knowledge regarding the optimal method for endometrial sampling to accurately determine the histological type and grade of endometrial cancer. This retrospective study assesses the diagnostic accuracy of endometrial sampling techniques. It compares the accuracy of different sampling methods, such as hysteroscopy, dilation and curettage (D&C), and endometrial biopsy, for detecting the histologic type and the grading of endometrial carcinoma.

## Materials and methods

We conducted a retrospective, single-center study to evaluate the diagnostic accuracy of endometrial sampling methods in women diagnosed with uterine carcinoma. A secondary objective was to compare the histological consistency of the methods (Pipelle biopsy, D&C, and hysteroscopy) with the final hysterectomy specimen. The study was approved by the Galilee Medical Center Institutional Review Board (NHR-0071-24), and informed consent was waived due to its retrospective nature.

The study included data from women who underwent a primary hysterectomy after an endometrial biopsy, and the final histology was malignancy at the Galilee Medical Center, Nahariya, Israel, between January 2010 and May 2024. 

Eligible patients underwent an initial preoperative endometrial sampling procedure, performed by Pipelle biopsy, D&C, or hysteroscopy, followed by hysterectomy within two months of the endometrial sampling. Women with other concurrent gynecological malignancies were excluded.

Patient data were retrospectively retrieved from the hospital’s medical records. The data collected included pre- and postoperative histological subtype and tumor grade, patient age, menopausal status, parity, indication for the endometrial sampling, and diagnostic sampling method. Cases with incomplete data were excluded from the analysis.

Patients were classified into three groups based on the initial sampling method used: the Pipelle biopsy group, the D&C group, and the hysteroscopy group.

Each case with two different sampling methods performed before the hysterectomy was considered a separate entry to assess the accuracy of each method independently.

Statistical analysis

Continuous variables exhibiting normal distributions were reported as means ± standard deviations, while median values and ranges were employed to describe variables that did not follow a normal distribution. Categorical variables were compared between groups using the Chi-square test or Fisher's exact test when expected frequencies were less than 5. Continuous variables were compared using the Mann-Whitney or independent t-test, contingent upon identifying a normal distribution. 

The diagnostic performance of the sampling techniques was assessed as the overall diagnostic accuracy, defined as the proportion of correct diagnoses. Additionally, we analyzed the agreement between preoperative histological diagnoses based on endometrial sampling and the final histopathological results from the surgical specimens using Kappa (κ) statistics, with 95% confidence intervals (CIs). The strength of agreement was categorized according to the following Kappa value ranges: <0, no agreement; 0-0.19, poor agreement; 0.20-0.39, fair agreement; 0.40-0.59, moderate agreement; 0.60-0.79, substantial agreement; 0.80-1, excellent agreement. A two-tailed p-value of <0.05 was considered statistically significant. A p-value of less than 0.05 was deemed statistically significant. Statistical analyses were conducted using IBM SPSS Statistics for Windows, version 25 (IBM Corp., Armonk, NY, USA).

## Results

This retrospective study included 188 women with a confirmed histopathological diagnosis of uterine cancer. The median age of the participants was 64.2 years (±11.5 years). Among the cases, 124 women (66.0%) were diagnosed with type 1 endometrial cancer, 36 (19.1%) with type 2 endometrial cancer, 22 (11.7%) had no malignancy, and 6 (3.1%) presented with other forms of uterine sarcoma. The median body mass index (BMI) was 33.5 (±7.6). Concerning biopsy indications, 139 women (73.9%) underwent endometrial sampling due to postmenopausal bleeding, 23 women (12.3%) due to abnormal uterine bleeding (AUB), and 26 women (13.8%) were asymptomatic. The histological type’s preoperative and postoperative diagnostic accuracy was 83.5% (157 women). Regarding grading accuracy, 68.6% (126 women) were consistent, with 49 cases (26.1%) upgraded and 10 cases (5.3%) downgraded (Table 1).

**Table 1:** Characteristics of the overall study population

Characteristic	N=188
Age, mean (±sd)	64.2 (±11.5)
BMI, mean (±sd)	33.5 (±7.6)
Smoking, n (%)	13 (6.9%)
Diabetes mellitus, n (%)	73 (38.8%)
Hyperlipidemia, n (%)	71 (37.8%)
Obesity, n (%)	38 (20.2%)
Parity, median (range)	2 (0-12)
Biopsy indication	
Asymptomatic, n (%)	26 (13.8%)
Abnormal uterine bleeding, n (%)	23 (12.3%)
Postmenopausal bleeding, n (%)	139 (73.9%)
Preoperation histology	
Non-malignant, n (%)	22 (11.7%)
Endometrial endometrioid carcinoma, n (%)	124 (66.0%)
Non-endometrial endometrioid carcinoma, n (%)	36 (19.1%)
Sarcoma, n (%)	6 (3.1%)
Post-operation histology	
Non-malignant, n (%)	2 (1.1%)
Endometrial endometrioid carcinoma, n (%)	134 (71.3%)
Non-endometrial endometrioid carcinoma, n (%)	41 (21.9%)
Sarcoma, n (%)	11 (5.8%)
Positive cytology	21 (14.2%)
Biopsy accuracy	
Histology accuracy, n (%)	157 (83.5%)
Grade accuracy, n (%)	129 (68.6%)
Upgrade, n (%)	49 (26.1%)
Downgrade, n (%)	10 (5.3%)

Regarding sampling methods, 78 women (41.5%) underwent hysteroscopy, 61 women (32.4%) had a Pipelle biopsy, and 49 women (26.1%) underwent D&C. Hysteroscopic biopsy showed significantly higher accuracy for histological type and grading than Pipelle biopsy: 91.0% vs. 75.4% for histological type (p=0.018) and 76.9% vs. 57.4% for grading accuracy (p=0.017). No significant differences between hysteroscopy and D&C in histological type and grading accuracy (91.0% vs. 81.6%, p=0.169; 76.9% vs. 69.4%, p=0.407). Similarly, no significant differences were observed between Pipelle biopsy and D&C (75.4% vs. 81.6%, p = 0.492; 57.4% vs. 69.4%, p = 0.236) (Table 2).

**Table 2:** Comparison of diagnostic accuracy among endometrial sampling methods; hysteroscopy, dilation and curettage (D&C), and Pipelle biopsy Comparisons were performed using the Chi-square test. Chi-square values for each comparison: histology (hysteroscopy vs. Pipelle: χ²=6.26; hysteroscopy vs. D&C: χ²=2.41; Pipelle vs. D&C: χ²=0.61) and grade (hysteroscopy vs. Pipelle: χ²=6.04; hysteroscopy vs. D&C: χ²=0.89; Pipelle vs. D&C: χ²=1.67). a-b: pairwise comparison of Hysteroscopy and Pipelle methods; b-c: pairwise comparison of Pipelle and D&C methods; a-c:  pairwise comparison of Hysteroscopy and D&C methods

Parameters	Endometrial sampling methods			p-values		
	Hysteroscopy N=78^a^	Pipelle N=61^b^	D&C N=49^c^	a-b	b-c	a-c
Histology accuracy, n (%)	71 (91.0%)	46 (75.4%)	40 (81.6%)	0.018	0.492	0.169
Grade accuracy, n (%)	60 (76.9%)	35 (57.4%)	34 (69.4%)	0.017	0.236	0.407

In assessing diagnostic concordance between preoperative endometrial biopsies and final hysterectomy findings, among 188 women, the overall histologic concordance was 83%, with 95.1% accuracy for endometrioid carcinoma cases (ECC), 91.6% for non-endometrioid carcinoma (non-ECC) cases, and 83.3% for sarcoma cases. The kappa value was 0.649 (Moderate); p < 0.001 (Table 3).

**Table 3:** Concordance between pathological findings from endometrial biopsy and subsequent hysterectomy kappa value, 0.649 (Moderate); diagnostic concordance 83% (156 out of 188 pairs); P < 0.001 ECC: endometrial endometroid carcinoma; non-ECC: non-endometrial endometroid carcinoma

Preoperative biopsy	n (%)	Postoperative				Concordance to hysterectomy, n (%)
		No malignancy	ECC	Non-ECC	Sarcoma	
No malignancy	22 (11.7%)	0	14	2	6	0
EEC	124 (65.9%)	1	118	5	0	118 (95.1%)
Non-ECC	36 (19.1%)	1	2	33	0	33 (91.6%)
Sarcoma	6 (3.3%)	0	0	1	5	5 (83.3%)
Total	188	2	134	41	11	156 (83%)

For Pipelle biopsy (61 patients), the concordance rate was 75.4%, with a kappa value of 0.441 (Weak); p < 0.001 (Table 4).

**Table 4:** Concordance between pathological findings from endometrial biopsy by Pipelle and subsequent hysterectomy Kappa value, 0.441 (weak); diagnostic concordance 75.4% (46 out of 61 pairs); P < 0.001 ECC: endometrial endometroid carcinoma; non-ECC: non-endometrial endometroid carcinoma

Preoperative biopsy	n (%)	Postoperative			Concordance to hysterectomy, n (%)
		ECC	Non-ECC	Sarcoma	
No malignancy	11 (18.0%)	8	1	2	0
EEC	41 (67.2%)	39	2	0	39 (95%)
Non-ECC	8 (13.1%)	2	6	0	6 (75%)
Sarcoma	1 (1.6%)	0	0	1	1 (100%)
Total	61	49	9	3	46 (75.4%)

For D&C (49 patients), concordance reached 83.7%, with a kappa value of 0.731 (Moderate); p < 0.001 (Table 5).

**Table 5:** Concordance between pathological findings from endometrial biopsy by D&C and subsequent hysterectomy Kappa value, 0.731 (Moderate); diagnostic concordance 83.7% (41 out of 49 pairs); P < 0.001 D&C: dilation and curettage; ECC: endometrial endometroid carcinoma; non-ECC: non-endometrial endometroid carcinoma

Preoperative biopsy	n (%)	Postoperative			Concordance to hysterectomy, n (%)
		ECC	Non-ECC	Sarcoma	
No malignancy	7 (14.2%)	2	1	4	0
EEC	27 (55.1%)	26	1	0	26 (96.2%)
Non-ECC	12 (24.4%)	0	12	0	12 (100%)
Sarcoma	3 (6.1%)	0	0	3	3 (100%)
Total	49	28	14	7	41 (83.7%)

For hysteroscopic biopsy (78 patients), concordance was 88.5%, with a kappa value of 0.729 (Moderate); p < 0.001 (Table 6).

**Table 6:** Concordance between pathological findings from endometrial biopsy by hysteroscopy and subsequent hysterectomy Kappa value, 0.729 (Moderate); diagnostic concordance 88.5% (69 out of 78 pairs); P < 0.001 ECC: endometrial endometroid carcinoma; non-ECC: non-endometrial endometroid carcinoma

Preoperative biopsy	n (%)	Postoperative				Concordance to hysterectomy, n (%)
		No malignancy	ECC	Non-ECC	Sarcoma	
No malignancy	4 (5.1%)	0	4	0	0	0
EEC	56 (71.7%)	1	53	2	0	53 (94.6%)
Non-ECC	16 (20.5%)	1	0	15	0	15 (93.7%)
Sarcoma	2 (2.5%)	0	0	1	1	1 (50%)
Total	78	2	57	18	1	69 (88.5%)

## Discussion

This study evaluates the diagnostic accuracy of different endometrial sampling methods for histologic subtyping and grading in endometrial cancer, emphasizing the comparative performance of hysteroscopy, Pipelle biopsy, and D&C. Our findings contribute to the ongoing discourse regarding preoperative diagnostic accuracy and the optimal choice of biopsy method for effective risk stratification and treatment planning in endometrial cancer.

Histologic and grading concordance and diagnostic accuracy

Our findings demonstrate an overall histologic concordance of 83% between preoperative endometrial sampling and final hysterectomy specimens. This aligns closely with previous studies that report histologic concordance rates in the 80-90% range for endometrial cancers [14,16]. Endometrioid carcinoma cases showed high accuracy at 95.1%, consistent with prior literature [12,13]. Non-endometrioid carcinoma concordance was slightly lower (91.6%), likely due to the tumors’ inherent histological diversity and higher-grade nature, which the literature also highlights as a challenge for accurate preoperative assessment [17].

Regarding tumor grading, our study observed a concordance of 68.6%, with 26.1% of cases upgraded and 5.3% downgraded following final surgical evaluation. This discrepancy between preoperative and postoperative grading aligns with earlier findings, which reveal that grading is frequently inconsistent due to the presence of mixed-grade patterns within tumors. One of the main reasons for disagreement on grade between endometrial sampling and final diagnosis could be the limited amount of tissue often obtained by preoperative endometrial sampling, which can lead to difficulties in assessing tumor grade. Consequently, there is insufficient tissue for diagnosis in 15%-68% of the samples [18-20]. This insufficiency rate differs per sampling method [21].

Biopsy method comparison

Our study indicated that hysteroscopic biopsy showed significantly higher accuracy for both histologic type (91.0%) and grading (76.9%) compared to Pipelle biopsy (75.4% and 57.4%, respectively; p = 0.018 and p = 0.017, Chi-square test). Literature similarly suggests that more comprehensive sampling methods, such as hysteroscopy, yield higher accuracy, as limited tissue samples from Pipelle biopsies often fail to capture heterogeneous or high-grade tumor regions, contributing to misclassification [14,22-24]. Our kappa values confirmed this pattern, with hysteroscopy demonstrating moderate concordance (κ = 0.729). In contrast, the Pipelle biopsy only showed weak concordance (κ = 0.441), underscoring the advantages of using hysteroscopy for diagnostic accuracy.

Pipelle biopsy, widely used, often yields smaller tissue samples, which may need to be revised for detecting high-grade or mixed lesions, thus explaining its lower accuracy rates observed here and in other studies [6,8]. Interestingly, D&C showed comparable hysteroscopy, although not statistically significant, affirming its utility as a more accurate option than Pipelle in settings where hysteroscopy is not available or feasible. These values underscore hysteroscopy’s diagnostic advantage in ensuring a higher level of agreement with final pathology, a finding corroborated by the literature, which suggests its superiority in cases requiring precise histologic subtyping and grading [14].

These findings highlight the need for careful consideration of the initial biopsy method in endometrial cancer cases, especially in patients at higher risk for high-grade or non-endometrioid tumors. Given the limitations of histologic sampling alone, especially in terms of accurate grading, integrating molecular markers as part of a multimodal preoperative assessment could enhance diagnostic accuracy, as recommended by recent studies [11,12]. The application of molecular profiling may help overcome discrepancies in histologic grading, reduce the likelihood of under- or overtreatment, and ultimately contribute to improved clinical outcomes in the management of endometrial cancer. 

The study's limitations include its single-center, retrospective design, which affects generalizability and may introduce potential selection bias, as it only included patients who underwent hysterectomy within two months of biopsy. Incomplete data from medical records and the lack of molecular profiling in diagnostic criteria are additional concerns. However, the study's strengths lie in its practical comparisons of endometrial sampling methods (hysteroscopy, D&C, and Pipelle biopsy) in real-world settings, utilizing histologic concordance with hysterectomy specimens as a standard to validate diagnostic accuracy. The study's large sample size and extended timeframe also enhance its statistical power and ability to track changes in clinical practice.

## Conclusions

The study highlights the moderate diagnostic accuracy of endometrial sampling in determining the histologic type and grade of endometrial cancer. Hysteroscopy demonstrated the highest accuracy for histologic subtype and grading. While D&C showed similar results to hysteroscopy, the Pipelle biopsy was less reliable due to its limited tissue sampling capacity, particularly in terms of grading accuracy. Although limited by its single-center, retrospective design and lack of molecular profiling, this study supports hysteroscopy as the preferred method where precise diagnosis is critical. These findings suggest that integrating molecular markers with histological assessment could improve diagnostic accuracy and patient outcomes in endometrial cancer care.
